# Reviewing progress: 7 year trends in characteristics of adults and children enrolled at HIV care and treatment clinics in the United Republic of Tanzania

**DOI:** 10.1186/1471-2458-13-1016

**Published:** 2013-10-27

**Authors:** Harriet Nuwagaba-Biribonwoha, Bonita Kilama, Gretchen Antelman, Ahmed Khatib, Annette Almeida, William Reidy, Gongo Ramadhani, Matthew R Lamb, Redempta Mbatia, Elaine J Abrams

**Affiliations:** 1 ICAP-Columbia University, Mailman School of Public Health, 535 W 116th Street, New York, NY, 10027, USA; 2 Department of Epidemiology, Columbia University, Mailman School of Public Health, 535 W 116th Street, New York, NY, 10027, USA; 3 National AIDS Control Program, P.O. BOX 11857, Dar es Salaam, United Republic of Tanzania; 4 Zanzibar AIDS Control Program, P. O. Box 236, Unguja Zanzibar, United Republic of Tanzania; 5 Centers for Disease Control, P.O. Box 9123, Dar es Salaam, Tanzania; 6 Tanzania Health Promotion Support, P.O. BOX 32605, Dar es Salaam, Tanzania

**Keywords:** ART program, HIV-infected adults, HIV-infected children, Trends at enrolment, Trends at ART initiation, Tanzania

## Abstract

**Background:**

To evaluate the on-going scale-up of HIV programs, we assessed trends in patient characteristics at enrolment and ART initiation over 7 years of implementation.

**Methods:**

Data were from Optimal Models, a prospective open cohort study of HIV-infected (HIV+) adults (≥15 years) and children (<15 years) enrolled from January 2005 to December 2011 at 44 HIV clinics in 3 regions of mainland Tanzania (Kagera, Kigoma, Pwani) and Zanzibar. Comparative statistics for trends in characteristics of patients enrolled in 2005–2007, 2008–2009 and 2010–2011 were examined.

**Results:**

Overall 62,801 HIV + patients were enrolled: 58,102(92.5%) adults, (66.5% female); 4,699(7.5%) children.

Among adults, pregnant women enrolment increased: 6.8%, 2005–2007; 12.1%, 2008–2009; 17.2%, 2010–2011; as did entry into care from prevention of mother-to-child HIV transmission (PMTCT) programs: 6.6%, 2005–2007; 9.5%, 2008–2009; 12.6%, 2010–2011

. WHO stage IV at enrolment declined: 27.1%, 2005–2007; 20.2%, 2008–2009; 11.1% 2010–2011. Of the 42.5% and 29.5% with CD4+ data at enrolment and ART initiation respectively, median CD4+ count increased: 210 cells/μL, 2005–2007; 262 cells/μL, 2008–2009; 266 cells/μL 2010–2011; but median CD4+ at ART initiation did not change (148 cells/μL overall). Stavudine initiation declined: 84.9%, 2005–2007; 43.1%, 2008–2009; 19.7%, 2010–2011.

Among children, median age (years) at enrolment decreased from 6.1(IQR:2.7-10.0) in 2005–2007 to 4.8(IQR:1.9-8.6) in 2008–2009, and 4.1(IQR:1.5-8.1) in 2010–2011 and children <24 months increased from 18.5% to 26.1% and 31.5% respectively. Entry from PMTCT was 7.0%, 2005–2007; 10.7%, 2008–2009; 15.0%, 2010–2011. WHO stage IV at enrolment declined from 22.9%, 2005–2007, to 18.3%, 2008–2009 to 13.9%, 2010–2011. Proportion initiating stavudine was 39.8% 2005–2007; 39.5%, 2008–2009; 26.1%, 2010–2011. Median age at ART initiation also declined significantly.

**Conclusions:**

Over time, the proportion of pregnant women and of adults and children enrolled from PMTCT programs increased. There was a decline in adults and children with advanced HIV disease at enrolment and initiation of stavudine. Pediatric age at enrolment and ART initiation declined. Results suggest HIV program maturation from an emergency response.

## Background

In the United Republic of Tanzania (Tanzania), HIV prevalence among adults aged 15–49 years was last estimated at 5.7% in 2008/2009 [1]. An estimated 1,400,000 people were living with HIV by 2010, of whom 200,000 are children under 15 years of age [2]. Approximately 610,000 persons living with HIV were in need of antiretroviral therapy (ART) [2] using the ART eligibility criteria in the 2006 World Health Organization (WHO) guidelines [3]. ART coverage was estimated at 42% among adults and 18% among children, and Tanzania was one of five countries (together with Nigeria, South Africa, Kenya, and Uganda) that contributed to 50% of the global unmet need for pediatric ART [4]. To address the HIV epidemic, Tanzania has been providing free HIV care and treatment, including antiretroviral therapy (ART) since 2004. Over 900 clinics provide ART in the country [5] and 258,069 patients were receiving ART by the end of 2010 [4].

The scale-up of ART services and the large number of patients receiving ART are important successes in Tanzania. An evaluation conducted in the early years of program implementation revealed that ART services had been introduced at 210 facilities within 3 years, but there was limited male and pediatric enrolment [5]. Like many sub-Saharan African countries, Tanzania’s HIV services were initiated at urban higher-level health facilities and prioritized patients with the most advanced HIV disease [6]. Over time, there have been efforts to encourage HIV testing, decentralize HIV services, expand prevention of mother-to-child HIV transmission (PMTCT) programs, increase the number of people receiving HIV care and treatment, and phase out the use of stavudine due to significant toxicity [7-9]. In order to inform the continuing scale-up of ART programs in this country, we examined trends over 7 years in characteristics of adults and children attending HIV care and treatment clinics in selected regions of Tanzania.

## Methods

### Setting

ICAP at Columbia University with funding from the United States President’s Emergency Plan for AIDS Relief (PEPFAR) provided programmatic, health facility and health systems support to Tanzania in the establishment of HIV care and treatment clinics (CTCs). ICAP’s support in Tanzania began in 2004 and was regionalized to Kagera, Kigoma (north-west Tanzania), Pwani (east coastal region), and the island of Zanzibar. In 2007–2008, HIV prevalence among adults aged 15–49 years in these regions was estimated at 5.3% in Pwani, 3.4% in Kagera, 0.9% in Kigoma and 0.6% in Zanzibar. By December 2011, a total of 127 CTCs had been supported in these regions and a total of 89,780 HIV-infected patients enrolled into HIV care [10]. ICAP support included training of health care providers, building capacity for sustainable clinical mentorship and supervision, and implementation of the national monitoring and evaluation (M&E) system for HIV care and treatment. The Identifying Optimal Models of Care and Treatment in sub-Saharan Africa (Optimal Models) study was funded by the Centers for Disease Control and Prevention (CDC) to analyze routinely collected patient and site-level data for program evaluation. Optimal Models is an open cohort, with patients entering and exiting according to their routine care.

### Routinely collected patient-level data

When patients attend HIV care and treatment clinics, demographic and clinical characteristics and treatment details e.g. age, gender, source of referral, WHO staging, CD4+ cell count (CD4+), ART status and regimen are recorded on their patient medical card. During the study observation period, ART initiation was according to national guidelines which aligned with the 2003 and 2006 WHO ART initiation guidelines [3,6,8,11]. The data were entered into a Ministry of Health Microsoft Access database, which is maintained at the site level, updated with every patient visit, and used for reporting to the national government and implementation partners. Implementation of the patient-level database in ICAP-supported regions was prioritized at clinics with the highest patient volume. By the end of 2011, the database was in use at 44 clinics and captured 70% of all patients ever enrolled across the 127 clinics. At the time of introduction of the database, all retrospective data were entered into the database. Quarterly supervision visits were conducted by ICAP in collaboration with Ministry of Health Regional and District Health Management Teams to support site-level data entry clerks in data quality assurance.

### Health facility data

Health facility and program characteristics were captured annually using a site assessment tool [12,13]. Data collected include the site characteristics e.g. location of the site and level of health facility (health centers and dispensaries were categorized as primary facilities; and district, missionary or regional referral hospitals as secondary. Program characteristics that describe the services provided (e.g. presence and location of Voluntary Counseling and Testing (VCT), PMTCT and CD4+ testing services) were also documented. Data from the most recent survey (2011) were used to give a descriptive snap-shot of site and program characteristics.

### Data analysis

The patient record data were de-identified and anonymized, then combined across health facilities to create an analytic dataset. The analysis (using SPSS 18) explored trends in patient characteristics among adults (≥15 years) and children (<15 years) enrolled from January 2005 to December 2011. Seven patients were missing age data and excluded from the analysis. WHO stage and CD4+ at enrolment and ART initiation, respectively, were based on records within < 90 days before and < 30 days after date of enrolment/ART initiation with priority given to data before and closest to the enrolment/ART initiation date. ART eligibility was defined using the 2006 WHO guidelines [14]. Descriptive comparative statistics were computed for differences in characteristics of patients enrolled in 2005–2007, 2008–2009 and 2010–2011. These time categories were estimated to represent particular phases of the ART program in Tanzania: the early start-up phase in 2005–2007 where ART services were introduced starting at major hospitals; the rapid scale-up and decentralization phase in 2008–2009 where ART was rolled out to a large number of clinics including lower level health facilities; and a maturation phase 2010–2011 where fewer clinics were newly implementing ART services and the focus for existing clinics was ensuring sustainability and enhancing service provision through support services. Differences and trends in patient characteristics between time categories were tested using chi-square tests for categorical variables and generating linear-by-linear association p values. Differences in medians over time groups were tested with the Jonckheere-Terpstra test, and for other nominal variables like gender and source of referral, the Kruskal-Wallis test was used. Adult trends in WHO stage and CD4+ were stratified by gender and pregnancy status. Missing data are an important focus area for data quality improvement, so for variables with >5% missing data, tables include an indication for data available for each variable with a test for trend over time.

### Ethical approval

Optimal Models involves analysis of de-identified routinely collected data and received non-human subjects research determination from Columbia University Institutional Review Board and the Centers for Disease Control. The study was also approved by Institutional Review Boards in mainland Tanzania and Zanzibar.

## Results

**Figure 1:**
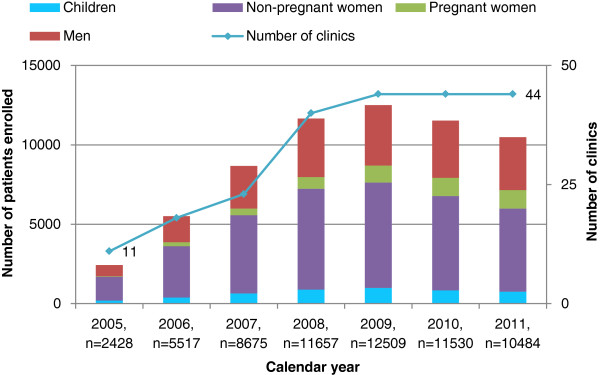
Enrolment of children and adults (men, pregnant women and non-pregnant women) by calendar year in Tanzania (2005–2011).

A total of 62,801 patients, 58,102 (92.5%) adults and 4,699 (7.5%) children, were enrolled at the 44 clinics, with peak enrolment in 2009 (Figure 1). The majority of patients (58.0%) and half the clinics were from Kagera region (Table 1). Most patients (80.9%) attended urban/semi-urban clinics, where 79.5% of the clinics were located. Nearly half of the patients (46.1%) were enrolled at the 29.5% public secondary health facilities, while 22.4% were enrolled at the 36.4% public primary health clinics. VCT and PMTCT services were available at all but 1 clinic, and all patients were enrolled at clinics that provided antenatal services and CD4+ testing on or off-site.

**Table 1:** Clinic and program characteristics, and number of adults and children ever enrolled at the study HIV care and treatment clinics in Tanzania (2011)

		**Clinics**		**Adults attending clinics**		**Children attending clinics**		**All patients attending clinics**	
		**N = 44**	**(%)**	**N = 58,102**	**(%)**	**N = 4,699**	**(%)**	**N = 62,801**	**(%)**
Region	Kagera	22	(50.0)	33952	(58.4)	2479	(52.8)	36431	(58.0)
	Kigoma	5	(11.4)	7194	(12.4)	714	(15.2)	7908	(12.6)
	Pwani	12	(27.3)	15453	(26.6)	1359	(28.9)	16812	(26.8)
	Zanzibar^1^	5	(11.4)	1503	(2.6)	147	(3.1)	1650	(2.6)
Location	Urban/Semi-Urban	35	(79.5)	46969	(80.8)	3830	(81.5)	50799	(80.9)
	Rural	9	(20.5)	11133	(19.2)	869	(18.5)	12002	(19.1)
Facility Level	Public Primary	16	(36.4)	13065	(22.5)	997	(21.2)	14062	(22.4)
	Public Secondary^2^	13	(29.5)	26679	(45.9)	2260	(48.1)	28939	(46.1)
	Private	15	(34.1)	18358	(31.6)	1442	(30.7)	19800	(31.5)
VCT services available at clinic	Yes	43	(97.7)	57590	(99.1)	4671	(99.4)	62261	(99.1)
PMTCT services available at clinic	Yes	43	(97.7)	57942	(99.7)	4689	(99.8)	62631	(99.7)
Availability of CD4+ testing	On-site	21	(47.7)	42292	(72.8)	3522	(75.0)	45814	(73.0)
	Off-site^*3*^	23	(52.3)	15810	(27.2)	1177	(25.0)	16987	(27.0)
Availability of infant diagnostic testing	DBS collected lab at another facility	42	(95.5)	57479	(98.9)	4644	(98.8)	62123	(98.9)
	Not available	2	(4.5)	623	(1.1)	55	(1.2)	678	(1.1)

^*1*^*Excludes data from the national referral hospital.*^*2*^*Includes 1 tertiary facility in Zanzibar.*^*3*^*Off-site means blood is collected at care and treatment clinic (CTC), CD4+ analyzed at a laboratory outside the health facility and the results returned to the CTC where they are picked by the patient.*

### Adult (≥15 years) characteristics

At enrolment, 66.5% were female and 12.5% of these were pregnant (Table 2). The overall median age was 35.2 years (yrs) (IQR 29.1-42.4). Median age of men (38.8 yrs, IQR 32.5-45.9) was significantly higher than non-pregnant women (33.4 yrs, IQR 27.7-40.2) and pregnant women (28.2 yrs, IQR 24.1-32.6). Half the adults (52.3%) were enrolled from VCT programs, 9.9% from PMTCT and 9.3% transferred in from other clinics. Overall, 84.4% adults had a WHO clinical stage recorded at enrolment, of whom 17.9% had advanced HIV disease with WHO stage IV. Among the 42.5% adults with documented CD4+ at enrolment, the overall median CD4+ was 251 cells/μL (IQR 104–463); and it was significantly higher among adults from PMTCT (432 cells/μL, IQR 254–620) compared with VCT (257 cells/μL, IQR 109–472) and other referral points (212 cells/μL, IQR 86–420); a difference that remained statistically significant after stratifying by gender and pregnancy status (data not shown). Only 29.5% of adults had documented CD4+ at ART initiation and median CD4+ was 148 cells/μL (IQR 67–236). Nearly half (49.2%) of the adults initiated ART across the 7 year period, and of these 58.2% were prescribed stavudine in the initial ART regimen.

**Table 2:** Characteristics at enrolment and ART initiation of adults attending HIV clinics Tanzania by calendar year group

		** All adults**		**Adult characteristics by year of enrolment**						
		** All years**		** 2005-2007**		** 2008-2009**		** 2010-2011**		**p value for trend**
		** N = 58,102**		** N = 15,392**		** N = 22,289**		** N = 20,421**		
		**n **	**(%) **	**n **	**(%) **	**n **	**(%) **	**n **	**(%) **	
Gender^1^	Male	19467	(33.5)	5033	(32.7)	7498	(33.6)	6936	(34.0)	0.02
	Female	38634	(66.5)	10358	(67.3)	14791	(66.4)	13485	(66.0)	
Females pregnant at enrolment^2^		4816	(12.5)	704	(6.8)	1793	(12.1)	2319	(17.2)	<0.0001
Age at enrolment (years)	15-19 years	1748	(3.0)	399	(2.6)	725	(3.3)	624	(3.1)	<0.0001
	20-29 years	14985	(25.8)	3416	(22.2)	5897	(26.5)	5672	(27.8)	
	30-39 years	22683	(39.0)	6165	(40.1)	8733	(39.2)	7785	(38.1)	
	10-49 years	12693	(21.8)	3838	(24.9)	4667	(20.9)	4188	(20.5)	
	50+ years	5993	(10.3)	1574	(10.2)	2267	(10.2)	2152	(10.5)	
Point of entry into HIV care	Point of entry documented	53739	(92.5)	13405	(87.1)	21027	(94.3)	19739	(92.5)	<0.0001
	VCT	28126	(52.3)	5035	(37.6)	11943	(56.8)	11148	(57.8)	<0.0001
	PMTCT	5311	(9.9)	887	(6.6)	1993	(9.5)	2431	(12.6)	
	Other^3^	20295	(37.8)	7483	(55.8)	7091	(33.7)	5721	(29.6)	
Transferred in	Yes	5398	(9.3)	643	(4.2)	2720	(12.2)	2035	(10.0)	<0.0001
WHO stage at enrolment	WHO stage documented	49048	(84.4)	9545	(62.0)	20018	(89.8)	19485	(95.4)	<0.0001
	I	12593	(25.7)	1615	(16.9)	4742	(23.7)	6263	(32.0)	<0.0001
	II	13309	(27.1)	1901	(19.9)	5117	(25.6)	6291	(32.3)	
	III	14368	(29.3)	3445	(36.1)	6121	(30.6)	4802	(24.6)	
	IV	8778	(17.9)	2584	(27.1)	4038	(20.2)	2156	(11.1)	
CD4+ at enrolment^4^	CD4+ documented	24713	(42.5)	6093	(39.6)	9804	(44.0)	8816	(43.2)	<0.0001
(cells/μL)	<50	3467	(14.0)	981	(16.1)	1294	(13.2)	1192	(13.5)	<0.0001
	50-99	2464	(10.0)	718	(11.8)	918	(9.4)	828	(9.4)	
	100-199	4494	(18.2)	1241	(20.4)	1737	(17.7)	1516	(17.2)	
	200-349	5113	(20.7)	1217	(20.0)	2044	(20.8)	1852	(21.0)	
	≥350	9175	(37.1)	1936	(31.8)	3811	(38.9)	3428	(38.9)	
ART eligibility at enrolment^5^	Eligible	18464	(31.8)	5273	(34.3)	7601	(34.1)	5590	(27.4)	<0.0001
	Not eligible	26151	(45.0)	4373	(28.4)	10059	(45.1)	11719	(57.4)	
	Unknown eligibility	13487	(23.2)	5746	(37.3)	4629	(20.8)	3112	(15.2)	
CD4+ at ART initiation^4^, (cells/μL)	CD4+ documented	17142	(29.5)	5086	(33.0)	6945	(31.2)	5111	(25.0)	<0.0001
	<50	3379	(19.7)	1020	(20.1)	1307	(18.8)	1052	(20.6)	<0.0001
	50-99	2597	(15.1)	803	(15.8)	982	(14.1)	812	(15.9)	
	100-199	5319	(31.0)	1638	(32.2)	2132	(30.7)	1549	(30.3)	
	200-349	4421	(25.8)	1255	(24.7)	1888	(27.2)	1278	(25.0)	
	≥350	1426	(8.3)	370	(7.3)	636	(9.2)	420	(8.2)	
Started ART	All adults who started ART	28578	(49.2)	9304	(60.4)	11164	(50.1)	8110	(39.7)	<0.0001
	Females who started ART^6^	18445	(64.5)	6190	(66.5)	7199	(64.5)	5056	(62.3)	<0.0001
	Pregnant at ART initiation^7^	504	(2.7)	111	(1.8)	211	(2.9)	182	(3.6)	<0.0001
First ART regimen^8^	Stavudine containing	16635	(58.2)	7900	(84.9)	7139	(63.9)	1596	(19.7)	<0.0001
	Zidovudine containing	11086	(38.8)	1338	(14.4)	3894	(34.9)	5854	(72.2)	
	Other^9^	857	(3.0)	66	(0.7)	131	(1.2)	660	(8.1)	

^*1*^*1 adult missing gender data.*^*2*^*Denominator for percentage is females enrolled.*^*3*^*I TB/HIV clinics, inpatient clinics, outpatient clinics, and Persons Living with HIV associations .*^*4*^*CD4+ at any date < 90 days before or < 30 days after date of enrolment/ART initiation with priority given to date before and closest to the enrolment/ART initiation date.*^*5*^*Based on 2006 WHO eligibility criteria.*^*6*^*Denominator for percentage is all adults who started ART.*^*7*^*Denominator for percentage is females who started ART.*^*8*^*Denominator for percentage is those who started ART, stavudine and zidovudine containing regimens were combined with lamivudine and nevirapine or efavirenz.*^*9*^*Includes less commonly used drugs like abacavir, tenofovir and protease inhibitors.*

### Trends in adult characteristics

Among females, the proportion pregnant at enrolment increased from 6.8% in 2005–2007 to 12.1% in 2008–2009 and 17.2% in 2010–2011 (p < 0.0001) (Table 2). Overall, adult median age at enrolment decreased modestly from 36.1 (IQR 30.0-43.1) in 2005–2007 to 35.0 (IQR 28.9-42.1) in 2008–2009 and 34.8 (IQR 28.5-42.1) in 2010–2011 (p < 0001). On stratifying by gender and pregnancy status, the decrease in median age over time only remained statistically significant among non-pregnant women (data not shown). VCT as a point of entry into care contributed 37.6% of HIV + adults in 2005–2007, 56.8% in 2008–2009 and 57.8% in 2010–2011, p < 0.0001. There were also increasing proportions of adults enrolled from PMTCT programs over time: 6.6% in 2005–2007; 9.5% in 2008–2009; and 12.6% in 2010–2011. The proportion of adults transferring in from other clinics increased from 4.2% in 2005–2007 to 12.2% in 2008–2009, then declined to 10.0% in 2010–2011, though the overall proportion was increasing over time, p < 0.0001.

**Figure 2:**
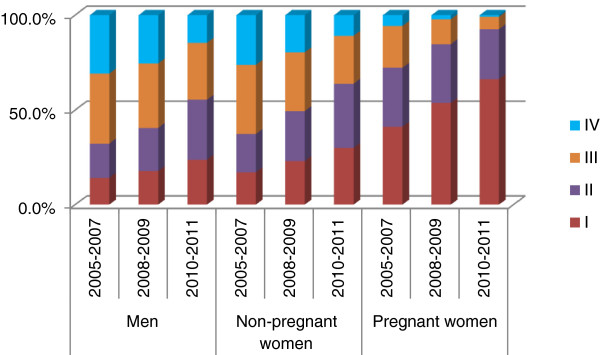
Changes over time in adult WHO stage at enrolment by gender and pregnancy status in Tanzania.

**Figure 3:**
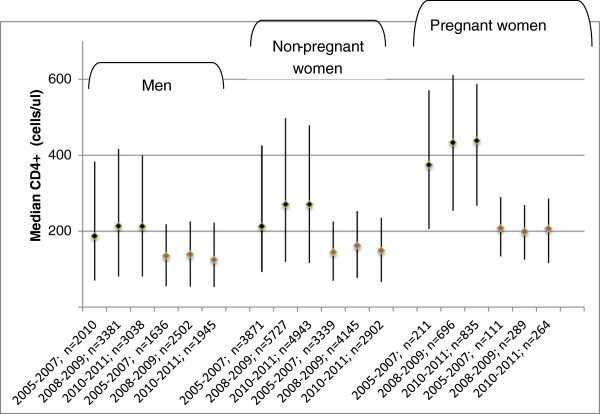
Changes over time in adult median CD4+ and inter-quartile range at enrolment and ART initiation by gender and pregnancy status.

There was an increase in WHO clinical stage documentation from 62.0%, 2005–2007 to 89.8%, 2006–2007, to 95.4%, 2010–2011 (p < 0.0001). The percentage of adults enrolled with WHO clinical stage IV declined from 27.1% in 2005–2007 to 20.2% in 2008–2009 and 11.1% in 2010–2011 (p < 0.0001). This decline was statistically significant among males as well as non-pregnant and pregnant women (Figure 2). Overtime, a slightly higher proportion of adults had CD4+ documentation at enrolment: 39.6%, 2005–2007; 44.0%, 2008–2009; 43.2%, 2010–2011, but there was a decline in CD4+ documentation at ART initiation 33.0%, 2005–2007; 31.2%, 2008–2009; 25.0%, 2010–2011, (p < 0.0001). In the sub-set of adults with CD4+, median CD4+ at enrolment increased from 210 cells/μL (IQR 88–418) in 2005–2007 to 262 cells/μL (IQR 111–484) in 2008–2009 and 266 cells/μL (IQR 112–468) in 2010–2011 (p < 0001). On stratifying by gender and pregnancy status, this increase was only statistically significant among the non-pregnant women (Figure 3). There were no statistically significant changes in median CD4+ at ART initiation even after stratifying by gender and pregnancy status (Figure 3). Median CD4+ at ART initiation was 143 cells/μL (IQR 65–225) in 2005–2007, 155 cells/μL (IQR 70–245) in 2008–2009 and 143 cells/μL (IQR 63–235) in 2010–2011, p = 0.6. There was a decline in stavudine use among the adults that initiated ART, 84.9%, 63.9% and 19.7% of those enrolled in 2005–2007, 2008–2009, and 2010–2011 respectively. A higher proportion of adults initiated AZT-containing regimens: 14.4%, 34.9% and 72.2% in the respective calendar year groups.

### Pediatric (<15 years) characteristics

Overall, 52.8% were female and median age at enrolment was 4.1 years (IQR 1.9-8.8), and at ART initiation 5.6 years (IQR 2.2-9.7). Main points of entry into care were VCT (44.4%) and PMTCT (11.2%); and 10.8% of children transferred in from other clinics (Table 3). The majority (85.5%) had documented WHO clinical stage at enrolment and 17.6% were stage IV. Nearly half (49.3%) were ≥5 years and of these, 40.6% and 28.5% had CD4+ at enrolment and ART initiation documented respectively. Median CD4+ at enrolment of children 5 years or older was 398 cells/μL (IQR 167–717) and at ART initiation 327 cells/μL (IQR 87–394). Overall, 55.3% initiated ART, of whom 35.4% were prescribed stavudine in the initial ART regimen.

**Table 3:** Characteristics at enrolment and ART initiation of children attending HIV clinics Tanzania by calendar year group

		** All children**		**Pediatric characteristics by year of enrolment**						
		** All years**		** 2005-2007**		** 2008-2009**		** 2010-2011**		**p value for trend**
		** N = 4,699**		** N = 1,229**		** N = 1,877**		** N = 1,593**		
		**n **	**(%) **	**n **	**(%) **	**n **	**(%) **	**n **	**(%) **	
Gender	Female	2479	(52.8)	674	(54.8)	984	(52.4)	821	(51.5)	0.09
	Male	2220	(47.2)	555	(45.2)	893	(47.6)	772	(48.5)	
Age at enrolment (years)	0-1 years	1219	(25.9)	227	(18.5)	491	(26.1)	501	(31.5)	<0.0001
	2-4 years	1162	(24.7)	280	(22.8)	479	(25.5)	403	(25.3)	
	5-9 years	1431	(30.5)	413	(33.6)	586	(31.2)	432	(27.1)	
	10-14 years	887	(18.9)	309	(25.1)	321	(17.1)	257	(16.1)	
Point of entry into HIV care	Point of entry documented	4294	(91.4)	1066	(86.7)	1757	(93.6)	1471	(92.3)	<0.0001
	VCT	1908	(44.4)	356	(33.4)	867	(49.3)	685	(46.6)	<0.0001
	PMTCT	483	(11.2)	75	(7.0)	188	(10.7)	220	(15.0)	
	Other ^1^	1903	(44.3)	635	(59.6)	702	(40.0)	566	(38.5)	
Transferred in	Yes	506	(10.8)	54	(4.4)	249	(13.3)	203	(12.7)	<0.0001
WHO stage at enrolment	WHO stage documented	4017	(85.5)	816	(66.4)	1695	(90.3)	1506	(94.5)	<0.0001
	I	836	(20.8)	110	(13.5)	351	(20.7)	375	(24.9)	<0.0001
	II	1183	(29.4)	189	(23.1)	477	(28.1)	517	(34.3)	
	III	1291	(32.1)	330	(40.4)	557	(32.9)	404	(26.8)	
	IV	707	(17.6)	187	(22.9)	310	(18.3)	210	(13.9)	
ART eligibility at enrolment^2^	Eligible	1936	(41.2)	468	(38.1)	810	(43.2)	658	(41.3)	<0.0001
	Not eligible	1447	(30.8)	284	(23.1)	607	(32.3)	556	(34.9)	
	Unknown eligibility	1316	(28.0)	477	(38.8)	460	(24.5)	379	(23.8)	
Started ART	Yes	2599	(55.3)	742	(60.4)	1045	(55.7)	812	(51.0)	<0.0001
Age at ART initiation (years)	0-1 years	690	(26.5)	108	(14.6)	270	(25.8)	312	(38.4)	<0.0001
	2-4 years	576	(22.2)	150	(20.2)	249	(23.8)	177	(21.8)	
	5-9 years	799	(30.7)	266	(35.8)	338	(32.3)	195	(24.0)	
	10-14 years	534	(20.5)	218	(29.4)	188	(18.0)	128	(15.8)	
First ART regimen^3^	Stavudine containing	920	(35.4)	295	(39.8)	413	(39.5)	212	(26.1)	<0.0001
	Zidovudine containing	1636	(62.9)	440	(59.3)	623	(59.6)	573	(70.6)	
	Other^4^	43	(1.7)	7	(0.9)	9	(0.9)	27	(3.3)	
CD4+ at enrolment^5^	***Number of children ≥5y***	***2318***	***-***	***722***	***-***	***907***	***-***	***689***	***-***	
(cells/μL), children ≥5 years	CD4+ documented	941	(40.6)	282	(39.1)	364	(40.1)	295	(42.8)	0.15
	<50	112	(11.9)	35	(12.4)	43	(11.8)	34	(11.5)	0.06
	50-99	50	(5.3)	23	(8.2)	11	(3.0)	16	(5.4)	
	100-199	111	(11.8)	39	(13.8)	48	(13.2)	24	(8.1)	
	200-349	134	(14.2)	39	(13.8)	53	(14.6)	42	(14.2)	
	≥350	534	(56.7)	146	(51.8)	209	(57.4)	179	(60.7)	
CD4+ at ART initiation^5^, (cells/μL), children ≥5 years	CD4+ documented	660	(28.5)	219	(30.3)	276	(30.4)	165	(23.9)	0.007
	<50	111	(16.8)	37	(16.9)	40	(14.5)	34	(20.6)	0.28
	50-99	68	(10.3)	28	(12.8)	24	(8.7)	16	(9.7)	
	100-199	138	(20.9)	43	(19.6)	67	(24.3)	28	(17.0)	
	200-349	149	(22.6)	55	(25.1)	58	(21.0)	36	(21.8)	
	≥350	194	(29.4)	56	(25.6)	87	(31.5)	51	(30.9)	

^*1*^*Includes TB/HIV clinics, inpatient clinics, outpatient clinics, and Persons Living with HIV associations.*^*2*^*Based on WHO 2006 guidelines.*^*3*^*As a percentage of those who started ART, stavudine and zidovudine containing regimens were combined with lamivudine and nevirapine or efavirenz.*^*4*^*Includes less commonly used drugs in the initial ART regimen like abacavir, tenofovir and protease inhibitors.*^*5*^*CD4+ done at any date < 90 days before or < 30 days after date of enrolment/ART initiation with priority given to date before and closest to the enrolment/ART initiation date.*

### Trends in pediatric characteristics

**Figure 4:**
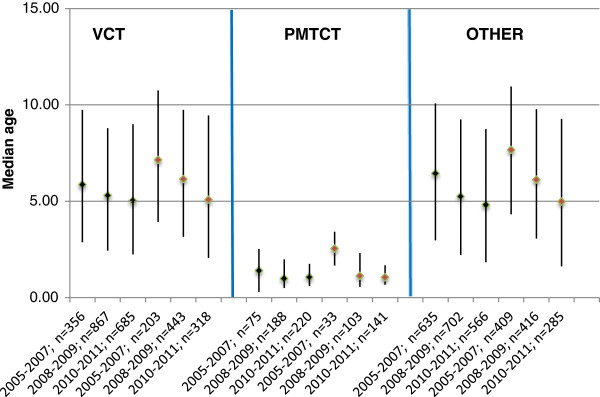
Changes over time in children’s median age and inter-quartile range at enrolment and ART initiation by source of referral to HIV clinics in Tanzania.

Although absolute numbers of children increased, there were no changes in the proportion of children over time: 7.4%, 2005–2007; 7.8%, 2008–2009; 7.4%, 2010–2011. The children’s median age at enrolment decreased from 6.1 yrs (IQR 2.7-10.0) in 2005–2007 to 4.8 yrs (IQR 1.9-8.6) in 2008–2009, and 4.1 yrs (IQR 1.5-8.1) in 2010–2011 (p < 0.0001). Children aged <24 months at enrolment increased from 18.5% in 2005–2007 to 26.1% in 2008–2009 and 31.5% in 2010–2011, p < 0.0001, (Table 3). Referrals from PMTCT clinics tended to be younger (Figure 4) and were an increasing proportion of children over time: 7.0%, 2005–2007; 10.7%, 2008–2009; 15.0%, 2010–2011, p < 0.0001, (Table 3). The proportion of children transferring in from other clinics increased from 4.4% in 2005–2007 to 13.3% in 2008–2009 then declined to 12.7% in 2010–2011, but an overall upward trend was observed (p < 0.0001).

There was an increase in WHO clinical stage documentation from 66.4%, 2005–2007 to 90.3%, 2006–2007, to 94.5%, 2010–2011. The percentage of children with WHO clinical stage IV declined from 22.9%, 2005–2007 to 18.3%, 2008–2009 to 13.9%, 2010–2011 (p < 0.0001). Among the 2,318 children ≥5 years, there was no statistically significant change in documentation of CD4+ at enrolment, and documentation of CD4+ at ART initiation declined over time (Table 3). In this subset of the 5 years and older children with CD4+ at enrolment (40.6%) and ART initiation (28.5%), median CD4+ at enrolment increased from 378 cells/μL (IQR 123–697) in 2005–2007 to 401 cells/μL (IQR 172–718) in 2008–2009 and 451 cells/μL (IQR 198–744) in 2010–2011 (p = 0.035), and median CD4+ at ART initiation was 208 cells/μL (IQR 81–350), 223 cells/μL (IQR 107–402) and 223 cells/μL (IQR 60–396) in the respective time periods (p = 0.34). Of the 2,599 children that initiated ART, 39.8%, 39.5% and 26.1% of those enrolled in 2005–2007, 2008–2009, and 2010–2011 respectively initiated stavudine-containing regimens (p < 0.0001). Median pediatric age at ART initiation declined from 7.4 yrs (IQR 4.0-11.1) in 2005–2007 to 5.5 yrs (IQR 2.3-9.3) in 2008–2009, and 3.7 yrs (IQR 1.4-8.1) in 2010–2011 (p < 0.0001). The proportion of children aged <24 months at ART initiation increased from 14.6% in 2005–2007 to 25.8% in 2008–2009 and 38.4% in 2010–2011 (p < 0.0001). Children enrolled from PMTCT programs initiated ART at a much younger age than those enrolled from VCT and other referral points (Figure 4).

## Discussion

We reviewed trends of adult and pediatric characteristics at enrolment and ART initiation over 7 years of implementing HIV care and treatment programs in the United Republic of Tanzania. Our findings suggest that increasingly, adults and children are being enrolled from PMTCT programs and with less advanced HIV disease. We found an increasing proportion of pregnant women enrolled, younger age at enrolment among adults, younger age at enrolment and ART initiation among children, and fewer adults and children starting stavudine in their initial ART regimen. However, fewer than half the patients had CD4+ documentation and there were no changes in median CD4+ at ART initiation among adults and children. Overall, a large number of adults and children accessed HIV care and treatment, demonstrating the successful establishment and expansion of HIV care and treatment programs in Tanzania. In the 3 ICAP supported regions of mainland Tanzania (Kagera, Kigoma and Pwani), these data from sites with electronic databases represent 43% of the adult population estimated to be HIV+ by December 2009, implying that a significant proportion of the population in these regions had accessed HIV services [15]. Observed trends suggest progression from an emergency response to more mature HIV programs.

Over time, PMTCT programs had an increasing contribution to adults and children entering HIV care. This is further evidenced by the significant increase in the proportion of women pregnant at enrolment and may be an indication that the scale up of PMTCT programs is having a positive impact on linkage into care and treatment. Though small, the proportion of women pregnant at ART initiation also increased over time. The largest contributor to adults and children entering HIV care was VCT, particularly after 2007. This could reflect the success of the National Voluntary HIV Testing Campaign conducted in 2007/2008 [1]. The population was encouraged to know their HIV status and subsequently access HIV care and treatment, and it is possible that there was increased population awareness of VCT in subsequent years. The proportion of patients transferring into the study clinics peaked in 2008–2009, a period associated with a rapid and peak increase in the number of clinics with ART services and decentralization [16]. The declining proportion of patients enrolled with less advanced HIV disease over time is an encouraging finding, and may be linked to more referrals from VCT and PMTCT clinics which are typically attended by healthier patients as observed in our analysis and by others [17,18]. It could also reflect maturation of the Tanzanian program which in the early years prioritized identification and ART initiation of the patients with the most advanced HIV disease as specified in the national guidelines [6].

There have been significant improvements in WHO stage documentation over time, but CD4+ documentation remains low, even among the most recently enrolled adults and children. As a result, ART eligibility at enrolment could not be determined for at least 1 in five patients in the study population (with the exception of the most recently enrolled adults). Considering the liberal window attached to the enrolment visit in our analysis (<90 days before and <30 days after date of enrolment), this is a concern. CD4+ testing is an essential complement to WHO staging in accurately diagnosing HIV disease progression [19,20], but missing data due to various program and patient level factors is a common challenge in African programs [21-23]. A review of clinic data from ICAP sites suggests that inadequate lab facilities, frequent stock-outs of reagents, and machine breakdown contribute to the lack of CD4+ data [24]. Newer technologies such as point-of-care testing and same day results for CD4+ [25] should be explored as a strategy to improve availability of CD4+ data.

Despite the limitations with CD4+ testing, a large number of patients initiated ART, and it is possible that due to limited resources, CD4+ testing was deliberately omitted where patients had advanced HIV disease. According to Tanzania and WHO guidelines used during the study period [3,6,8,11], patients with clinically advanced HIV disease (WHO stage III or IV) could initiate ART without a CD4+ cell count. In further analyses of our study population, 84% of adults without CD4+ documentation at ART initiation were classified as WHO stage III or IV, suggesting that patients missing CD4+ cell counts were more likely to have advanced disease than patients receiving CD4+ testing. Consequently, the median CD4+ at ART initiation observed in our study is likely an overestimate of population CD4+ at ART initiation if the less clinically ill patients preferentially had CD4+ testing done.

We observed a modest increase in median CD4+ at enrolment over time comparable to other sub-Saharan Africa HIV programs [26,27], and suggesting healthier populations accessing care. Our data showed no statistically significant increase in median CD4+ at ART initiation, which could imply that greater efficiency is needed in the ART initiation process, or that clinics remain overwhelmed by the very ill patients in need of ART. This finding was unlike observations in other similar programs [26,28,29], but even in these programs, the absolute increase in median CD4+ at ART initiation was small. In Tanzania, implementation of changes in the ART initiation guidelines increasing the CD4+ threshold from 200 to 350 cells/μL began in 2010/2011, and this may contribute to the lack of change in our study observation period ending 2011. Our CD4+ trend data should however be cautiously interpreted due to the significant proportion of missing data. Further analyses of Optimal Models data to explore time to, and predictors of, timely ART initiation are planned.

There was a decline in stavudine use in the initial ART regimen, particularly significant among adults. This is in line with national and international guidelines that have advocated for the phasing out of this drug due to toxicities [7,14]. It is important that additional analyses continue to monitor this trend and also report the proportion of patients currently using the drug. Stavudine was not as widely used in initial pediatric-ART regimens and the decline in use was less dramatic compared to adults. This may be because in such resource-limited settings, inadequate availability of pediatric ART formulations often limits treatment choices [30].

One in three adults enrolled were male, and this did not change significantly over time. Evaluations conducted in other regions of Tanzania and Africa [4,31,32] have reported limited male involvement, but in Tanzania, this proportion is in line with the general population where males comprise 37% of those HIV infected [1]. Still, the impact of innovative strategies to encourage male access to HIV services, such as those explored in PMTCT programs [33-36] should be examined in this setting. The proportion of children in the study population did not increase significantly over time. This in a country with over 80% unmet need for pediatric ART [4] is a concerning finding and implies that efforts to diagnose and enroll children into ART care should be strengthened. Tanzania in collaboration with ICAP and other development partners is facilitating scaling up early infant diagnosis and creation of pediatric and adolescent friendly services [37-39]. This may explain our data showing younger age and less advanced children’s HIV disease at enrolment over time, perhaps pointing to successful early testing and linkage to care strategies implemented within PMTCT and VCT services. It is also possible that potential increase in the proportion of enrolled children expected from improved case-finding of pregnant women and children through scale-up of PMTCT services, early infant diagnosis programs and other pediatric focused initiatives was leveled out by more successful PMTCT programs resulting in fewer HIV-infected children.

The strengths of Optimal Models lie in the large sample of HIV-infected adults and children, and a variety of sites represented from multiple regions of Tanzania over a long observation period. The data are collected from ‘real-world’ settings: clinics and facilities where patients received care, giving insight to grass roots activities. This analysis serves as a baseline, and additional analyses evaluating critical implementation issues are planned. A limitation of this analysis was inadequate data to determine which clinics transferred-in patients were coming from, possibly overestimating the overall number enrolled if patients transferred within the 44 study sites. However, the impact of this overestimation is likely minimal since patients could potentially transfer-in from over 900 other clinics in Tanzania and beyond. Further review of the data indicated that transferring out of the study clinics did not proportionally increase over time: it declined from 17.9% to 13.1% to 5.9% in the respective calendar year groups.

This analysis did not control for site-level effects when testing for trends, however the entire patient population at the study sites was used. Whereas the trends are indicative of observations at the 44 study sites, the data cannot be considered as applying to all clinics in Tanzania more generally. Another limitation with this analysis pertains to data quality barriers generally associated with routine program data [40-43]. For example, it may well be that CD4+ testing was done for more patients but not captured in the patient records or the database. Routine data quality assessments are ongoing to continuously improve the data and minimize missing and erroneous data. These data encouragingly show improvements in data completeness for WHO stage and point of entry over time, and there were minimal missing data for some key variables like gender and age at enrolment.

## Conclusions

Over the early 7 years of HIV care and treatment program implementation in Tanzania, there was an increase in enrolment of pregnant women and adults and children enrolled from PMTCT programs. The proportion of adults and children with advanced HIV disease at enrolment declined, as did the stavudine use in the initial ART regimen. Median pediatric age at enrolment and ART initiation also declined. A large number of adults and children were enrolled into HIV care and initiated ART, demonstrating successful expansion of HIV services in the country. However, many patients were missing documentation of CD4+ at enrolment and ART initiation, an aspect that will require focus in further program enhancements.

## Consent

This study was based on de-identified routinely collected data. There was no interaction with patients for study purposes. The protocol received non-human subjects research designation and consent waiver under 45 CFR46. As such, individual consent was not sought for participating individuals.

## Competing interests

The authors declare that they have no competing interests.

## Authors’ contributions

HNB, BK, GA, AK, AAA, GR, RM, WR, EJA contributed to the conception of the analysis idea; HNB, BK, GA, AK, AAA, WR, ML, RM, contributed to data collection and cleaning; ML merged and coded the data; ML, GA, AAA advised on the analysis; HNB conducted the analyses and wrote the manuscript; BK, GA, AK, AAA, WR, GR, ML, RM, EJA, reviewed and contributed to the manuscript, EJA gave overall technical oversight for the analytic process and manuscript writing. All authors read and approved the final manuscript.

## Pre-publication history

The pre-publication history for this paper can be accessed here:

http://www.biomedcentral.com/1471-2458/13/1016/prepub
